# Nerve conduction features may serve as a diagnostic clue for neuronal intranuclear inclusion disease

**DOI:** 10.1093/braincomms/fcae221

**Published:** 2024-06-26

**Authors:** Kang-Yang Jih, Min-Yu Lan, Yi-Hong Liu, Yu-Shuen Tsai, Po-Yu Lin, Kuan-Lin Lai, Yi-Chu Liao, Yi-Chung Lee

**Affiliations:** Department of Neurology, Taipei Veterans General Hospital, Taipei 112, Taiwan; Department of Neurology, National Yang Ming Chiao Tung University School of Medicine, Taipei 112, Taiwan; Department of Physiology, National Yang Ming Chiao Tung University School of Medicine, Taipei 112, Taiwan; Department of Neurology, Chang Gung Memorial Hospital and Chang Gung University College of Medicine, Kaohsiung 833, Taiwan; Center for Parkinson’s Disease, Chang Gung Memorial Hospital and Chang Gung University College of Medicine, Kaohsiung 833, Taiwan; Department of Neurology, Taipei Veterans General Hospital, Taipei 112, Taiwan; Department of Neurology, National Yang Ming Chiao Tung University School of Medicine, Taipei 112, Taiwan; Cancer and Immunology Research Center, National Yang Ming Chiao Tung University, Taipei 112, Taiwan; Department of Neurology, National Cheng Kung University Hospital, College of Medicine, National Cheng Kung University, Tainan 704, Taiwan; Department of Neurology, Taipei Veterans General Hospital, Taipei 112, Taiwan; Department of Neurology, National Yang Ming Chiao Tung University School of Medicine, Taipei 112, Taiwan; Department of Neurology, Taipei Veterans General Hospital, Taipei 112, Taiwan; Department of Neurology, National Yang Ming Chiao Tung University School of Medicine, Taipei 112, Taiwan; Brain Research Center, National Yang Ming Chiao Tung University, Taipei 112, Taiwan; Department of Neurology, Taipei Veterans General Hospital, Taipei 112, Taiwan; Department of Neurology, National Yang Ming Chiao Tung University School of Medicine, Taipei 112, Taiwan; Brain Research Center, National Yang Ming Chiao Tung University, Taipei 112, Taiwan; Center for Intelligent Drug Systems and Smart Bio-devices (IDS2B), National Yang Ming Chiao Tung University, Hsinchu 300, Taiwan

**Keywords:** neuronal intranuclear inclusion disease, NIID, nerve conduction study, MNCV slowing

## Abstract

Neuronal intranuclear inclusion disease is a neurodegenerative disorder with a wide phenotypic spectrum, including peripheral neuropathy. This study aims to characterize the nerve conduction features and proposes an electrophysiological criterion to assist the diagnosis of neuronal intranuclear inclusion disease. In this study, nerve conduction studies were performed in 50 genetically confirmed neuronal intranuclear inclusion disease patients, 200 age- and sex-matched healthy controls and 40 patients with genetically unsolved leukoencephalopathy. Abnormal electrophysiological parameters were defined as mean values plus or minus two standardized deviations of the healthy controls or failure to evoke a response on the examined nerves. Compared to controls, neuronal intranuclear inclusion disease patients had significantly slower motor and sensory nerve conduction velocities, as well as lower amplitudes of compound motor action potentials and sensory nerve action potentials in all tested nerves (*P* < 0.05). Forty-eight of the 50 neuronal intranuclear inclusion disease patients (96%) had at least one abnormal electrophysiological parameter, with slowing of motor nerve conduction velocities being the most prevalent characteristic. The motor nerve conduction velocities of median, ulnar, peroneal and tibial nerves were 44.2 ± 5.5, 45.3 ± 6.1, 37.3 ± 5.3 and 35.6 ± 5.1 m/s, respectively, which were 12.4–13.6 m/s slower than those of the controls. The electrophysiological features were similar between neuronal intranuclear inclusion disease patients manifesting with CNS symptoms and those with PNS-predominant presentations. Thirteen of the 14 patients (93%) who underwent nerve conduction study within the first year of symptom onset exhibited abnormal findings, indicating that clinical or subclinical peripheral neuropathy is an early disease marker of neuronal intranuclear inclusion disease. We then assessed the feasibility of using motor nerve conduction velocity as a diagnostic tool of neuronal intranuclear inclusion disease and evaluated the diagnostic performance of various combinations of nerve conduction parameters using receiver operating characteristic curve analysis. The criterion of having at least two nerves with motor nerve conduction velocity ranging from 35 to 50 m/s in median/ulnar nerves and 30–40 m/s in tibial/peroneal nerves demonstrated high sensitivity (90%) and specificity (99%), with an area under the curve of 0.95, to distinguish neuronal intranuclear inclusion disease patients from healthy controls. The criterion’s diagnostic performance was validated on an independent cohort of 56 literature reported neuronal intranuclear inclusion disease cases (area under the curve = 0.93, sensitivity = 87.5%, specificity = 99.0%), and in distinguishing neuronal intranuclear inclusion disease from genetically unresolved leukoencephalopathy cases (sensitivity = 90.0%, specificity = 80.0%). In conclusion, mildly to moderately decreased motor nerve conduction velocity in multiple nerves is a significant electrophysiological hallmark assisting the diagnosis of neuronal intranuclear inclusion disease, regardless of CNS- or PNS-predominant manifestations.

## Teaser

Jih *et al*. reported that reduced motor nerve condition velocity is a significant and common feature of neuronal intranuclear inclusion disease regardless of its clinical phenotypes. The electrophysiological feature helps identify patients that warrant further genetic analysis of *NOTCH2NLC* GGC repeat expansions.

## Graphical Abstract

**Graphical Abstract:**
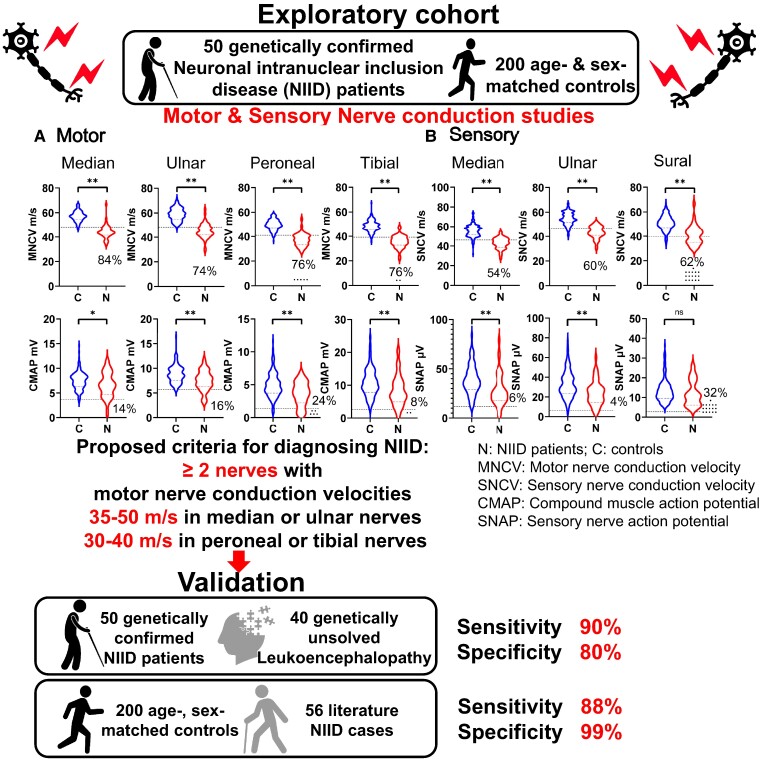

## Introduction

Neuronal intranuclear inclusion disease (NIID), caused by GGC repeat expansions in the 5′ UTR of the *NOTCH2NLC* gene, is a neurodegenerative disease with diverse clinical manifestations.^1-4^ Previous studies have categorized patients with NIID into three groups based on their major phenotypic features: dementia, parkinsonism and muscle weakness subtypes.^3,5,6^ Later studies reported a much more diverse clinical spectrum of NIID, encompassing paroxysmal symptoms such as seizure and acute encephalitis-like episode, oculopharyngodistal myopathy, ataxia, autonomic dysfunction,^4,7-9^ amyotrophic lateral sclerosis^10,11^ and essential tremor.^12,13^ Ubiquitin- and p62-positive eosinophilic intranuclear inclusions are the pathological hallmark of NIID. This characteristic feature has been found in neuronal and glial cells of both the central and peripheral nervous systems, as well as cells of many other types of tissue including stomach, urinary, bladder, lung and blood vessels.^7,14,15^ Extra neurological involvement, including the respiratory system, urinary system and digestive system, have been reported in both pathological studies and clinical observations.^3,8^

As a result, diagnosis of NIID is challenging since the symptoms overlap with many other common neurological diseases and display multisystem involvement. Several studies have noticed that peripheral neuropathy, including overt weakness/numbness at distal limbs and subclinical neuropathy is only evident by electrophysiological studies and could be a common feature of NIID.^3,4,7^ In one study comprising 247 NIID patients, muscle weakness and sensory disturbances were reported in less than a quarter of the patients. However, among those patients, 90.9% of the 110 cases who underwent nerve conduction studies showed abnormal motor and/or sensory nerve conduction.^4^ More importantly, these nerve conduction abnormalities were universally observed regardless of whether the clinical phenotypes were parkinsonism, dementia, limbs weakness or paroxysmal symptoms.^4^ In addition, subclinical electrophysiological abnormalities have been found in 96.4% of the NIID patients primarily manifesting with leukoencephalopathy.^16^

Although abnormal nerve conduction studies are common in NIID, the electrophysiological features and diagnostic value for patients with NIID remains unclear. In this study, we aimed to investigate if abnormal nerve conduction features could be used as a screening tool to assist the diagnosis of NIID. We first characterized the peripheral nerve conduction parameters of 50 NIID patients, and then evaluated the diagnostic accuracy of various criteria that combined different nerve conduction parameters. These criteria were used to differentiate NIID patients from the 200 age- and sex-matched healthy controls. The optimal criterion was selected based on receiver operating characteristic curve analysis. The diagnostic performance of this criterion was further validated by using both 56 literature reported cases of NIID as an independent cohort and 40 patients with genetically unsolved leukoencephalopathy as disease controls.

## Materials and methods

### Subjects

**Figure 1:**
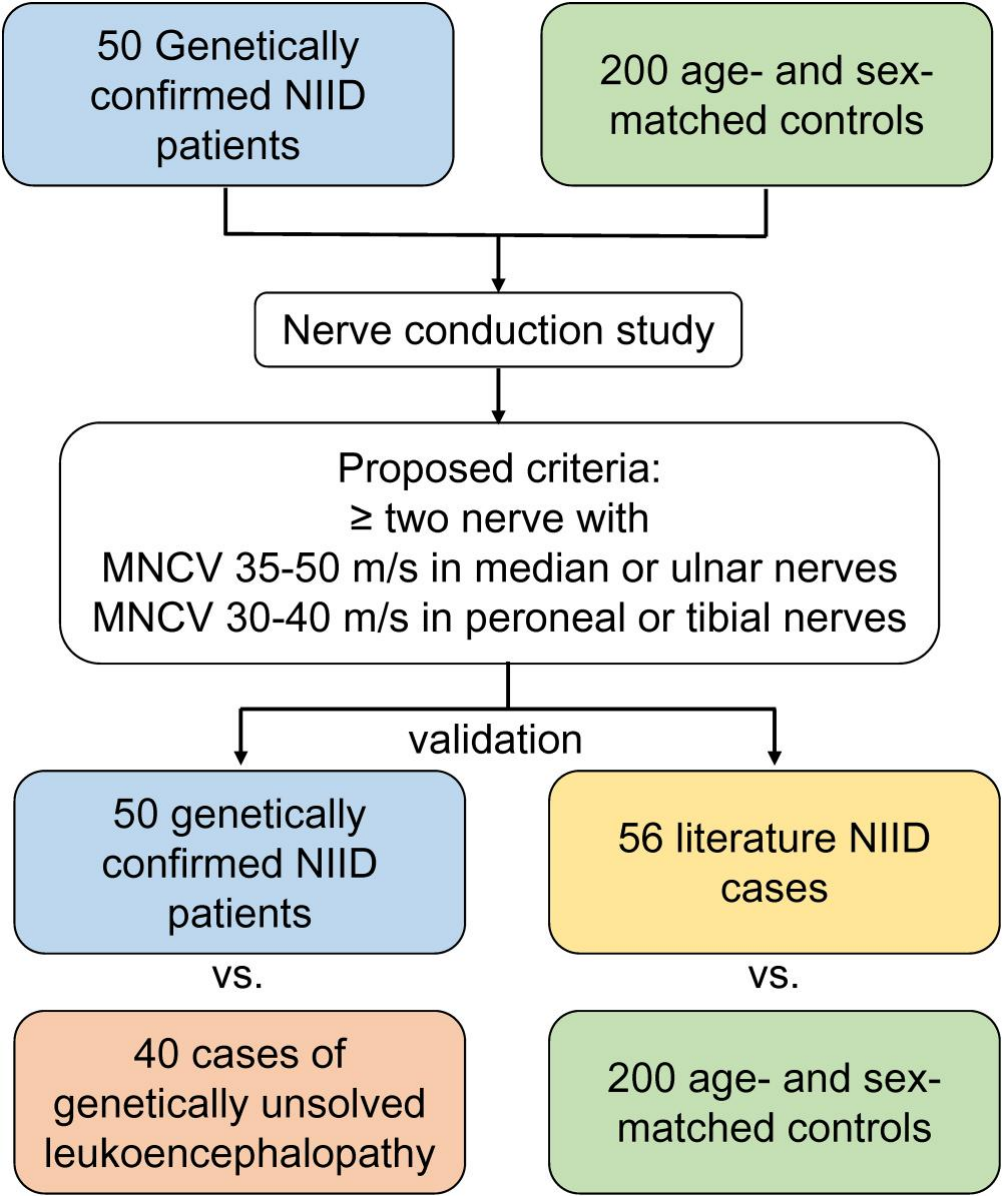
**Flowchart of study design and participants enrolment**. Nerve conduction studies from 50 genetically confirmed neuronal intranuclear inclusion disease (NIID) patients and 200 age- and sex-matched controls were analysed to develop a proposed criterion for NIID diagnosis. The criterion was further validated using 40 genetically unsolved leukoencephalopathy as negative controls and 56 literature reported NIID cases as positive controls. MNCV, motor nerve conduction velocity.

A consecutive series of 50 genetically confirmed NIID patients were enrolled from Taipei Veterans General Hospital, which is a tertiary medical centre accepting both self-referred patients and referrals from other hospitals in Taiwan. Clinical information and demographic data were collected, including age of onset, gender, initial presentations and associated symptoms. Patients with *NOTCH2NLC* GGC repeat expansions were divided into two groups: (i) a CNS-predominant group for subjects presenting with cognitive dysfunction, acute encephalitis-like episode, parkinsonism, or ataxic gait; and (ii) a PNS-predominant group for subjects presenting with muscle weakness or sensory symptoms. We also recruited 200 healthy control individuals who were age- and sex-matched to the 50 NIID patients by a 4:1 ratio (Fig. 1). We further reviewed the literature reporting NIID patient cohorts and collected the nerve conduction data of 56 genetically confirmed NIID cases from six studies as an independent cohort to validate the diagnostic performance of nerve conduction studies.^4,17-21^

To test whether the nerve conduction studies may help to distinguish NIID patients from subjects with leukoencephalopathy, 40 patients with genetically unsolved leukoencephalopathy were included who had (i) severe white matter hyperintensity in the deep white matter defined as Fazekas scale^22^ grade 3 on fluid-attenuated inversion recovery images; (ii) no genetic diagnosis after receiving targeted resequencing data covering 183 genes related to leukodystrophies and small vessel diseases (Supplementary Table 1); (iii) no *NOTCH2NLC* GGC repeat expansion; (iv) no cerebral infarct, lacunes or intracerebral haemorrhage lesions; and (v) no secondary causes of leukoencephalopathy, such as demyelinating disorders, toxin, infection or neoplasm.

### Genetic analysis for the GGC repeat expansion in *NOTCH2NLC*

The GGC repeat expansion in the 5′ UTR of *NOTCH2NLC* was analysed using fragment analysis and repeat-primed PCR as previously described.^2^ In brief, PCR-amplification of the GGC repeat region was performed with one primer being fluorescently labelled. Fragment length analysis of the fluorescently labelled amplicons was performed using a 3730xl Genetic Analyzer (Thermo Fisher Scientific, MA, USA) with GeneMapper software (v3.7) (Thermo Fisher Scientific). Human genome hg38 was used as the reference to calculate the GGC repeat numbers. Repeat-primed PCR was also performed to confirm the presence or absence of the *NOTCH2NLC* GGC repeat expansion.^2^ The amplicons generated by the repeat-primed PCR containing the expanded GGC repeats were analysed for the characteristic sawtooth pattern seen on resulting electropherograms.

### Electrophysiological studies

Nerve conduction studies were performed by standardized techniques in the 50 NIID patients, 200 healthy control subjects and 40 patients with genetically unsolved leukoencephalopathy. The Sierra Summit electromyography (Cadwell, Seattle, WA, USA) with surface electrodes was used for stimulation and recordings. MNCV, compound muscle action potential amplitudes (CMAP) and F wave latencies of median, ulnar, peroneal and tibial nerves were obtained, and sensory nerve conduction velocities (SNCV) and sensory nerve action potential amplitudes (SNAP) of median, ulnar and sural nerves were recorded as well. Results of the electrophysiological parameters on the non-dominant limbs were selected for analysis.

We defined SNCV, MNCV, SNAP and CMAP as abnormal results when these values were less than the mean minus two standardized deviations (SD) of the data from the 200 healthy controls or failure to evoke a response on the examined nerves. For F wave latencies, values longer than the mean plus two SD of the data from the 200 healthy controls and those unable to evoke a response were defined as abnormal results.

### Statistical analysis

IBM SPSS Statistics version 22 was used for statistical analysis. A two-tailed *P* value < 0.05 was considered statistically significant. All data were presented as *n* (%) or mean ± SD. Categorical variables were compared using the Chi-square test or Fisher’s exact test. Continuous variables were compared using the Student’s *t*-test.

Electrophysiological features of nerve conduction studies were compared between NIID patients and healthy controls, as well as NIID patients with CNS-predominant presentations and those with PNS-predominant presentations. Electrophysiological parameters that distinguished NIID patients from healthy controls with the highest area under the curve (AUC) value on the receiver operating characteristic curve analysis were selected. The corresponding sensitivity, specificity, positive predictive value and negative predictive value were calculated. The diagnostic performance of the proposed criterion using the electrophysiological data of the 56 literature reported NIID cases was further validated. The diagnostic performance of the criterion to distinguish between the 50 NIID patients and 40 patients with genetically unsolved leukoencephalopathy was also evaluated.

### Standard protocol approvals, registrations and patient consents

The study protocol has been approved by the institutional review boards of Taipei Veterans General Hospital, Taipei, Taiwan (Institutional Review Board No. 2021-02-005B). Informed consent was obtained from all study participants.

## Results

### Clinical characteristics of the patients with NIID

The demographic data and clinical features of the study participants are described in Table 1. The average size of the expanded *NOTCH2NLC* GGC repeats for the 50 NIID patients was 117.5 (range, 69–233), while the average repeat size of the wild-type alleles was 17.3 (range, 4–56). Two NIID patients had ultra-long GGC repeats, the exact number of which could not be measured accurately by fragment analysis. The mean age of symptom onset was 54.3 ± 14.3 years and the average age at nerve conduction studies was 60.2 ± 12.7 years. Thirty-five of the 50 NIID patients presented with CNS-predominant manifestations with the remaining 15 patients had PNS-predominant presentations.

**Table 1:** Clinical characteristics and demographic data of the study participants

Mean ± standard deviation (range) or *N* (%)	Pt with *NOTCH2NLC* GGC expansion (*N* = 50)	Pt with genetically undiagnosed leukoencephalopathy (*N* = 40)	Healthy controls (*N* = 200)
Male:female	16:34	20:20	64:136
Age of onset (years)	54.3 ± 14.3	65.4 ± 13.8	
Age at NCS (years)	60.2 ± 12.7	68.0 ± 13.0	59.8 ± 12.8
Disease duration at exam (years)	5.8 (0–31)	NA	
*NOTCH2NLC* GGC repeats			
Wide-type allele	17.3 ± 8.3 (4–56)	15.6 ± 5.9 (7–37)	ND
Expanded allele	117.5 ± 32.9 (69–233)^a^		ND
Initial symptoms			
CNS-predominant manifestations	35 (70.0%)	38 (95.0%)	
Cognitive impairment	12 (24.0%)	14 (35.0%)	
Acute encephalitis-like episode	6 (12.0%)	1 (2.5%)	
Parkinsonism	8 (16.0%)	1 (2.5%)	
Gait ataxia	9 (18.0%)	2 (5.0%)	
Acute stroke	0 (0.0%)	20 (55.0%)	
PNS-predominant manifestations	15 (30.0%)	2 (5.0%)	
Muscle weakness	6 (12.0%)	2 (5.0%)	
Sensory symptoms	9 (18.0%)	0 (0.0%)	

CNS, central nervous system; NCS, nerve conduction study; NA, not available; ND, not done; PNS, peripheral nervous system; Pt, patients.

^a^Two patients showed ultra-long GGC repeats, the exact number of which cannot be measured accurately by fragment analysis.

### Electrophysiological features of the patients with NIID

**Figure 2:**
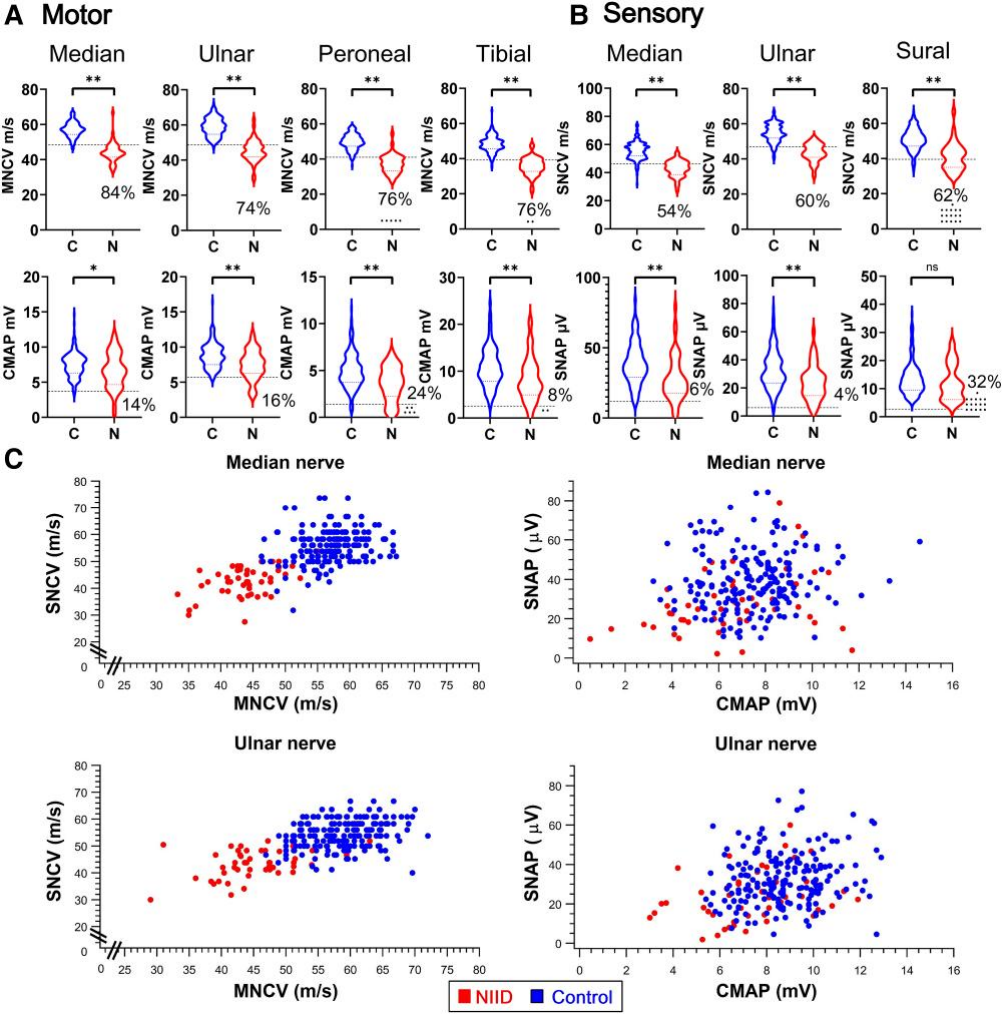
**Electrophysiological features of NIID patients and controls**. Violin plots showing motor (**A**) and sensory (**B**) nerve conduction velocity (upper row) and action potential (lower row) of controls (C, *n* = 200 subjects) and NIID patients (*N*, *n* = 50 subjects). Student’s *t*-test was used for statistical analysis. *P < 0.05, **P < 0.01. The *t* values of median, ulnar, peroneal and tibial MNCV are 15.3, 14.5, 14.6 and 15.7, respectively, and for CMAP are 2.3, 4.1, 4.4 and 3.5, respectively. The *t* values of median and ulnar SNCV are 13.7 and 13.8, respectively, and for SNAP are 4.4 and 4.4, respectively. The *t* value of sural SNCV is 6.9. Subjects with unprovokable response at the motor or sensory nerves were presented as small dots at the bottom of the violin plots. (**C**) The distribution of sensory nerve conduction velocities (SNCV) versus motor nerve conduction velocities (MNCV), as well as sensory nerve action potential amplitudes (SNAP) versus compound muscle action potential amplitudes (CMAP) in the median and ulnar nerves of the NIID patients (red dots) and controls (blue dots).

The findings of nerve conduction studies of the study participants are summarized in Fig. 2 and Table 2. Except for the sural nerve, NIID patients had significantly slower MNCV and SNCV, prolonged F responses, reduced CMAP and lower SNAP in all tested nerves compared to healthy controls (*P* < 0.05). The average MNCV of the median, ulnar, peroneal and tibial nerves for the 50 NIID patients was 44.2 ± 5.5, 45.3 ± 6.1, 37.3 ± 5.3 and 35.6 ± 5.1 m/s, respectively. The average MNCV of the NIID patients was significantly reduced in all examined nerves by 12.4–13.6 m/s compared to healthy controls (*P* < 0.001). Unlike the most common hereditary neuropathy, Charcot–Marie–Tooth 1A, MNCV of the NIID patients in the upper and lower limbs rarely fell below 35 m/s and 30 m/s, respectively (Table 3). Only 2% of the NIID patients had MNCV that fell below 35 m/s in the median and ulnar nerves, and 4–8% of the NIID patients had MCV < 30 m/s in the peroneal and tibial nerves. In addition, NIID patients also had significantly slower SNCV and lower amplitudes of CMAP and SNAP in all tested nerves compared to healthy controls (*P* < 0.05, Fig. 2 and Table 2).

**Table 2:** Comparison of the electrophysiological features between the patients carrying *NOTCH2NLC* GGC repeat expansion and healthy controls

Nerve/parameters	Mean ± SD or number (%)		Patients with abnormal values (%)^a^		
	Control (*N* = 200)	NIID (*N* = 50)	Overall (*N* = 50)	CNS-pred (*N* = 35)	PNS-pred (*N* = 15)
Median nerve					
MNCV (m/s)	57.1 ± 4.3	44.2 ± 5.5**	42 (84%)	30 (85.7%)	12 (80.0%)
CMAP (mv)	7.5 ± 1.7	6.6 ± 2.5*	7 (14%)	5 (14.2%)	2 (13.3%)
F wave latency (ms)	25.6 ± 1.9	31.8 ± 3.6**	39 (78%)	25 (71.4%)	14 (93.3%)
Absent F wave	0 (0%)	3 (6%)*			
SNCV (m/s)	55.5 ± 6.2	42.8 ± 5.7**	27 (54%)	22 (62.8%)	5 (33.3%)
SNAP (μv)	38.9 ± 14.9	27.9 ± 16.0**	3 (6%)	1 (2.9%)	2 (13.3%)
Ulnar nerve					
MNCV (m/s)	58.9 ± 5.1	45.3 ± 6.1**	37 (74%)	23 (65.7%)	14 (93.3%)
CMAP (mv)	8.8 ± 1.7	7.5 ± 2.1**	8 (16%)	6 (17.1%)	2 (13.3%)
F wave latency (ms)	25.8 ± 2.0	32.3 ± 3.7**	35 (70%)	22 (62.9%)	13 (86.7%)
Absent F wave	0 (0%)	1 (2%)*			
SNCV (m/s)	54.7 ± 5.2	43.5 ± 5.1**	30 (60%)	20 (57.1%)	10 (66.7%)
SNAP (μv)	32.7 ± 13.6	24.0 ± 12.4**	2 (4%)	1 (2.9%)	1 (6.7%)
Peroneal nerve					
MNCV (m/s)	49.7 ± 4.4	37.3 ± 5.3**	38 (76%)	28 (80.0%)	10 (66.7%)
CMAP (mv)	4.9 ± 1.8	3.6 ± 1.8**	12 (24%)	8 (22.9%)	4 (26.7%)
F wave latency (ms)	45.9 ± 3.9	56.2 ± 7.1**	35 (70%)	24 (68.6%)	11 (73.3%)
Absent F wave	1 (0.5%)	10 (20%)**			
Tibial nerve					
MNCV (m/s)	48.2 ± 4.6	35.6 ± 5.1**	38 (76%)	28 (80.0%)	10 (66.7%)
CMAP (mv)	10.8 ± 4.2	8.3 ± 4.7**	4 (8%)	3 (8.6%)	1 (6.7%)
F wave latency (ms)	46.8 ± 4.0	58.2 ± 7.3**	39 (78%)	26 (74.3%)	13 (86.7%)
Absent F wave	0 (0.0%)	4 (8%)*			
Sural nerve					
SNCV (m/s)	51.0 ± 6.1	41.1 ± 8.0**	31 (62%)	23 (65.7%)	8 (53.3%)
SNAP (μv)	13.5 ± 5.8	12.2 ± 6.5	16 (32%)	10 (28.6%)	6 (40.0%)
Not evoked	0 (0.0%)	16 (34.8%)**			

CMAP, compound muscle action potential; CNS-pred, central nervous system-predominant manifestations; MNCV, motor nerve conduction velocity; NIID, neuronal intranuclear inclusion disease; PNS-pred, peripheral nervous system-predominant manifestations; SNCV, sensory nerve conduction velocity; SNAP, sensory nerve action potential.

^a^Abnormal values were defined as values less than mean − 2 SD of the 200 normal controls on the examined nerves and unable to evoke a response in MNCV, SNCV, CMAP and SNAP, or values longer than mean + 2 SD of the normal controls on the examined nerves and unable to evoke a response in F wave latency.

*P value between 0.001 and 0.05 or ** *P* < 0.001 when comparing between NIID patients and healthy controls by Student’s *t*-test.

**Table 3:** Distribution of motor nerve conduction velocities (MNCV) of median, ulnar, peroneal and tibial nerves in the patients with neuronal intranuclear inclusion disease (NIID) in this study

	Number of nerves examined (%)			
	Median nerve	Ulnar nerve	Peroneal nerve	Tibia nerve
Not evoked	0 (0%)	0 (0%)	5 (10%)	2 (4%)
MNCV < 30 m/s	0 (0%)	1 (2%)	2 (4%)	4 (8%)
30 m/s ≤ MNCV < 35 m/s	1 (2%)	1 (2%)	14 (28%)	18 (36%)
35 m/s ≤ MNCV < 40 m/s	7 (14%)	5 (10%)	13 (26%)	15 (30%)
40 m/s ≤ MNCV < 45 m/s	24 (48%)	20 (40%)	14 (28%)	9 (18%)
45 m/s ≤ MNCV < 50 m/s	13 (26%)	12 (24%)	1 (2%)	2 (4%)
MNCV ≥ 50 m/s	5 (10%)	11 (22%)	1 (2%)	0 (0%)

We then defined the mean ± 2 SD cut-off values for the electrophysiological data reflecting abnormal results, using the 200 healthy controls and those unable to evoke a response. We found the majority of the NIID patients had abnormal electrophysiological findings in multiple nerves (Table 2). The percentages of NIID patients with abnormal MNCV ranged from 74% to 84% in the four tested nerves (median, ulnar, peroneal and tibial nerves), while the percentages of patients with abnormal CMAP amplitudes in the four tested nerves were 8% to 24%. Similar findings were also observed in three tested sensory nerves (median, ulnar and sural nerves), as the percentages of patients with abnormal SNCV (54–62%) were much higher than those with decreased SNAP amplitudes (4–32%). This suggests that slowing of conduction velocities, rather than reduced CMAP/SNAP amplitudes, may be a more sensitive marker for distinguishing NIID patients from healthy controls (Fig. 2C).

The electrophysiological features were similar between the NIID patients with CNS- and those with PNS-predominant presentations (Table 2, Supplementary Table 2). There was no statistically significant difference in the percentages of patients with abnormal electrophysiological parameters between the two groups (Table 2). These findings suggest that nerve conduction abnormalities are a common phenomenon in NIID patients regardless of their major clinical presentations. Furthermore, the severity of MNCV abnormality was not associated with the size of the expanded *NOTCH2NLC* GGC repeats and did not correlate with the age at examination (Supplementary Fig. 1). We further investigated whether disease duration could be a factor related to the percentages of patients with abnormal nerve conduction profiles. Our study revealed that 93% of the NIID patients had abnormal nerve conduction findings within the first year of symptom onset (Supplementary Table 3), indicating that peripheral nerve involvement occurs early in the disease course of NIID.

### Motor and sensory nerves were both affected in majority of NIID patients

To investigate whether there was any predominance in motor or sensory involvement in NIID neuropathy, we further analysed the number of nerves with abnormal MNCV or SNCV in each NIID patient (Table 4). The patients were defined as ‘pure motor’ involvement if the patient had at least one nerve with abnormal MNCV but none with abnormal SNCV. Conversely, ‘pure sensory’ involvement patients had normal MNCV and at least one abnormal SNCV. Patients were considered to have ‘mixed motor and sensory’ involvement if the patient had at least one nerve with abnormal MNCV and at least one nerve with abnormal SNCV. Two patients had normal MNCV and SNCV in all tested nerves while 42 of the 50 NIID patients (84%) showed abnormal conduction velocities in both motor and sensory nerves. Only four patients (8%) and two patients (4%) had pure motor and pure sensory involvement, respectively. These findings suggest that both motor and sensory nerves are affected in the majority of NIID patients.

**Table 4:** Electrophysiological characteristics of the patients with neuronal intranuclear inclusion disease (NIID) in the present study

	Number of patients with abnormal MNCV					
Number of patients with abnormal SNCV	0 nerve	1 nerve	2 nerves	3 nerves	4 nerves	Total
0 nerve	2	1	0	0	3	6
1 nerve	2	0	6	2	5	15
2 nerves	0	0	1	6	8	15
3 nerves	0	0	0	4	10	14
Total	4	1	7	12	26	50
Number of patients with abnormal MNCV/SNCV^a^						
Normal						2
Pure motor involvement (only abnormal MNCV)						4
Pure sensory involvement (only abnormal SNCV)						2
Mixed sensorimotor involvement						42

^a^Abnormal MNCV (motor nerve conduction velocity)/SNCV (sensory nerve conduction velocity) were defined as values less than mean − 2 SD of the 200 normal controls on the examined nerves or unable to evoke a response in MNCV or SNCV.

However, we found that motor and sensory components were not consistently affected in individual nerves among the patients with NIID. Taking the median nerve as example, 27 (54%) of the 50 NIID patients exhibited abnormal MNCV and SNCV in the median nerve, 15 (30%) only had abnormal median MNCV, none showed abnormal median SNCV alone, and 8 patients (16%) showed normal results in both tests **(**Supplementary Table 4**).** Similar findings were also observed in the ulnar nerve, where 25 patients (50%) demonstrated both abnormal MNCV and SNCV, and 17 patients (34%) displayed abnormal findings in either MNCV or SNCV. The inconsistent involvement of motor and sensory components in individual nerves may suggest that NIID patients carry the characteristics of neuronopathy that are different from typical polyneuropathy that presents with simultaneous involvement of the motor and sensory components in one individual nerve.

### Testing the feasibility of using MNCV as a diagnostic tool of NIID

Because MNCV slowing was the most prevalent electrophysiological abnormality in the 50 NIID patients, we proposed MNCV ranging from 35 to 50 m/s in the upper limbs and 30 to 40 m/s in the lower limbs as a potential criterion to diagnose NIID. The upper bounds, 50 m/s in the upper limbs and 40 m/s in the lower limbs, were selected because they are close to the cut-off value of abnormal MNCV (i.e. mean − 2 SD of the data from the 200 healthy controls) and could be easily used in clinical settings.^23^ The lower bounds of 35 m/s in the upper limbs and 30 m/s in the lower limbs were selected because very few NIID patients (<5%) had MNCV lower than these two values (Table 3).

We first tested the diagnostic performance of this proposed criterion in each nerve by comparing the percentages of subjects fulfilling this standard; when comparing the 50 NIID patients and 200 control subjects (Table 5). The proposed criterion of abnormal MNCV (35–50 m/s in the upper limbs and 30–40 m/s in the lower limbs) in any of the median, ulnar, peroneal and tibial nerves had a high specificity (95.5–99%) and moderate sensitivity (64–88%) to differentiate NIID patients from healthy controls. The AUCs ranged from 0.82 to 0.92 for each of the four nerves. We then combined the findings of multiple nerves and assessed the diagnostic performance of another two criteria: (i) any two of the median, ulnar, peroneal and tibial nerves fitting the abnormal MNCV standard; or (ii) any three of the four nerves fitting the abnormal MNCV standard. We found that the criterion of ‘any two nerves with abnormal MNCV’ yielded the highest AUC value (0.95) and provided an excellent specificity (99%) and sensitivity (90%) to differentiate between NIID patients and healthy controls (Table 5).

**Table 5:** Using electrophysiological features as a diagnostic tool to identify patients carrying *NOTCH2NLC* GGC repeat expansion

Patients fit the criteria, *N* (%)			Diagnostic accuracy (AUC; Sen, Spe, PPV, NPV)	
NIID in this study	NIID in literatures	Controls in this study	NIID in this study versus controls	NIID in the literatures versus controls
MNCV 35–50 m/s in the median nerve			AUC = 0.92	AUC = 0.81
45 (90.0%)	40 (71.4%)	9 (4.5%)	88.0%,95.5%,83.0%,97.0%	65.5%,95.5%,80.0%,91.0%
MNCV 35–50 m/s in the ulnar nerve			AUC = 0.88	AUC = 0.91
39 (78.0%)	43 (76.8%)	2 (1.0%)	76.0%,99.0%,95.0%,94.3%	82.7%,99.0%,95.6%,95.7%
MNCV 30–40 m/s in the peroneal nerve			AUC = 0.82	AUC = 0.87
34 (68.0%)	49 (87.5%)	2 (1.0%)	64.0%,99.0%,94.1%,91.7%	73.2%,99.0%,95.3%,93.0%
MNCV 30–40 m/s in the tibial nerve			AUC = 0.84	AUC = 0.83
39 (78.0%)	47 (83.9%)	6 (3.0%)	70.0%,97.0%,85.4%,92.8%	69.8%,97.0%,86.0%,92.4%
Any two nerves fulfilling the criteria			AUC = 0.95	AUC = 0.93
45 (90.0%)	49 (89.5%)	2 (1.0%)	90.0%,99.0%,95.7%,97.5%	87.5%,99.0%,96.1%,96.6%
Any three nerves fulfilling the criteria			AUC = 0.86	AUC = 0.85
38 (76.0%)	42 (75.0%)	1 (0.5%)	72.0%,99.5%,97.3%,93.4%	66.7%,99.5%,97.3%,91.7%

AUC, area under curve in receiver operating characteristic curve analysis; MNCV, motor nerve conduction velocity; NIID, neuronal intranuclear inclusion disease; NPV, negative predictive value; PPV, positive predictive value; Sen, sensitivity; Spe; specificity.

To further validate the diagnostic accuracy of this criterion in distinguishing NIID patients from healthy controls, the same analysis was performed using the electrophysiological data of 56 NIID patients from previous studies (Table 5).^4,17-21^ Forty-nine (89.5%) of the 56 literature reported cases fulfilled the criterion, ‘any two nerves with MNCV of 35–50 m/s in the upper limbs and 30–40 m/s in the lower limbs’. The test also showed a good diagnostic performance with a high AUC value (0.93) and corresponding sensitivity, specificity, positive predictive value and negative predictive value of 87.5%, 99.0%, 96.1% and 96.6%, respectively.

Since leukoencephalopathy is one of the major features of NIID,^24^ we further tested whether this criterion could faithfully distinguish the 50 NIID patients in our cohort from the 40 patients with genetically unsolved leukoencephalopathy. A significantly higher proportion of subjects had at least two nerves with abnormal MNCV in the NIID patients than in the patients with leukoencephalopathy (90.0% versus 20.0%, *P* < 0.001) (Table 6). The proposed criterion demonstrated good accuracy for distinguishing patients with NIID from patients with other leukoencephalopathies with corresponding sensitivity, specificity, positive predictive value and negative predictive values of 90.0%, 80.0%, 84.9% and 86.5%, respectively.

**Table 6:** Using electrophysiological features as a diagnostic tool to distinguish NIID patients from patients with genetically unsolved leukoencephalopathy

Patients fit the criteria, *N* (%)		Diagnostic accuracy				
NIID in this study	Genetically unsolved leukoencephalopathy	AUC	Sen	Spe	PPV	NPV
MNCV 35–50 m/s in the median nerve						
45 (90.0%)	6 (15.0%)	0.83	88.0%	85.0%	88.0%	85.0%
MNCV 35–50 m/s in the ulnar nerve						
39 (78.0%)	4 (10.0%)	0.87	76.0%	90.0%	90.5%	75.0%
MNCV 30–40 m/s in the peroneal nerve						
34 (68.0%)	9 (22.5%)	0.71	64.0%	77.5%	78.0%	63.3%
MNCV 30–40 m/s in the tibial nerve						
39 (78.0%)	10 (25.0%)	0.73	70.0%	75.0%	77.8%	66.7%
Any two nerves fulfilling the criteria						
45 (90.0%)	8 (20.0%)	0.85	90.0%	80.0%	84.9%	86.5%
Any three nerves fulfilling the criteria						
38 (76.0%)	5 (12.5%)	0.80	72.0%	87.5%	87.8%	71.4%

AUC, area under curve in receiver operating characteristic curve analysis; MNCV, motor nerve conduction velocity; NIID, neuronal intranuclear inclusion disease; NPV, negative predictive value; PPV, positive predictive value; Sen, sensitivity; Spe, specificity.

## Discussion

In this study, we delineate the nerve conduction features of patients with NIID and propose an electrophysiological criterion to facilitate the diagnosis of NIID. There are four major findings with important implications for the diagnostic criteria. Firstly, presentation of abnormal nerve conduction studies is a common feature among patients with NIID, regardless of whether they have CNS-predominant or PNS-predominant manifestations. Secondly, among the different electrophysiological parameters, the most prevalent signature was slowing of MNCV, followed by slowing of SNCV, prolonged F wave, reduced SNAP and decreased CMAP. However, the vast majority of the NIID patients had only a mild degree of MNCV slowing. Only 2% of the patients having median nerve MNCV < 35 m/s and 4% having ulnar nerve MNCV < 35 m/s. This suggests that the peripheral neuropathy in NIID may not be primarily attributed to a demyelination-predominant pathology. Thirdly, in contrast to the homogenous slowing of MNCV in all peripheral nerves as seen in patients with Charcot–Marie–Tooth disease type 1A, NIID patients showed an inconsistent and uneven involvement in the four tested nerves (i.e. median, ulnar, peroneal and tibial nerves). Additionally, we observed that one-third of the NIID patients had either motor or sensory involvement when analysing individual nerves, suggesting that the motor and sensory components of peripheral nerves may not be equally affected in NIID. Lastly, the present study confirms that nerve conduction studies may serve as a valuable tool to facilitate the diagnosis of NIID. The criterion we proposed of ‘having at least two nerves with MNCV of 35–50 m/s in the upper limbs and 30–40 m/s in the lower limbs’, demonstrated good sensitivity and specificity in distinguishing both NIID patients from normal controls and patients with genetically unsolved leukoencephalopathies. Nerve conduction studies are easily accessible and non-invasive, making the proposed criterion highly valuable in assisting the diagnosis of NIID, especially when symptoms overlap with those of many other neurodegenerative diseases.

Abnormal electrophysiological findings have consistently been reported in patients with NIID. MNCV or SNCV slowing have been observed in more than 90% of NIID patients, irrespective of their clinical presentations.^4,5,16^ In a study analysing 28 NIID patients with CNS symptoms, 96% had electrophysiological abnormalities, even in the absence of peripheral neuropathy symptoms.^16^ Another study revealed that ∼85% of NIID patients with dementia or parkinsonism phenotypes exhibited MNCV slowing.^5^ A more recent study further supported these findings, and showed the percentages of MNCV slowing were comparable among NIID patients presenting with dementia, movement disorders, muscle weakness or paroxysmal symptoms (87%, 86%, 94% and 92%, respectively).^4^ These results collectively suggest that peripheral neuropathy is a common feature observed in NIID patients across various phenotypes.

Our study demonstrated that 96% of the NIID patients had at least one abnormal electrophysiological parameter in the nerve conduction studies (Table 4). Moreover, 13 of the 14 (93%) patients exhibited abnormal electrophysiological findings within the first year of symptom onset, indicating that clinical or subclinical peripheral neuropathy could serve as an early disease marker for NIID (Supplementary Table 3). These findings indicate that damage of peripheral nerves is not only common in NIID but also occurs in the early disease stage. This highlights the potential of using nerve conduction study as a screening tool for NIID throughout different stages of the disease. It also raises an interesting question of whether damage to the peripheral nerves occurs before the onset of clinical symptoms. Exploring the role of nerve conduction studies in pre-clinical NIID carriers may hold significant importance as it could be utilized as a monitoring tool to track disease onset and progression. Further pathological investigations, such as nerve biopsy and immunohistological analysis, could provide insights into the pre-clinical pathological change of NIID neuropathy. In addition, recent advancements in diagnostic fluid biomarkers^25^ from peripheral nerve damage offer an opportunity to identify small molecule biomarkers associated with ubiquitous neuropathy among NIID patients.

Although abnormal electrophysiological findings can be detected in 96% of NIID patients, the electrophysiological feature of NIID-related neuropathy showed inconsistent and uneven involvement of the motor and sensory components in peripheral nerves. Only 50% of the patients exhibit both SNCV and MNCV slowing in the medium or ulnar nerve concomitantly (Supplementary Table 4). Furthermore, the severities of slowing of MNCV or SNCV and reduction of CMAP or SNAP amplitudes were observed to vary among the tested nerves (Fig. 2, Table 2). This phenomenon suggested that NIID-related neuropathy is unlikely caused by length-dependent axonal degeneration or homogenous demyelination, which are commonly seen in other types of polyneuropathies such as diabetes polyneuropathy or Charcot–Marie–Tooth disease 1A. The unique electrophysiological feature of NIID neuropathy cannot be explained solely by axonal loss or demyelination. Based on these unique electrophysiological properties, we hypothesized that NIID-related neuropathy may be partially derived from neuronopathy, in addition to the demyelinating or axonal pathology.^14^ As a result, the severity of damage depends on the extent of neuron loss that varies between each affected nerves. This hypothesis could explain the uneven nerve involvement of NIID neuropathy and the absence of length-dependent damage. This notion is supported by the presence of the pathognomonic intranuclear inclusions in spinal motor neurons and dorsal root ganglia of the NIID patients, which indicates injuries to neuronal cell bodies.^3^ However, the true cause of NIID-related neuropathy may be more complicated. The ubiquitin- and p62-positive intranuclear inclusions were also found in Schwann cells.^7^ Furthermore, sural nerve biopsies of NIID patients revealed not only demyelinating changes such as loss of myelinating fibres and thinly myelinating fibres^14,16^ but also axonal loss with significantly reduced density of both large and small fibres.^14^ These findings suggest that NIID-related neuropathy may result from a variable combination of neuronal damage and myelin defects.

In the present study, MNCV slowing is the most remarkable feature of NIID-related neuropathy that distinguish NIID from control subjects. The reason that MNCV are a better diagnostic clue than CMAP is that MNCV is the least variable electrophysiological parameter in the general population across a wide range of age groups (Fig. 2).^26,27^ In contrast, CMAP and SNAP exhibit greater differences between individuals that are not unrelated to NIID pathology.

Diagnosing NIID is challenging due to widely variable clinical presentations. The most common clinical features of NIID, including cognitive impairment, movement disorders and muscle weakness are non-specific for an accurate diagnosis. For patients with leukoencephalopathy, the presence of corticomedullary junction curvilinear hyperintense lesions on diffusion weight images has been demonstrated as a useful clue for diagnosing NIID.^24^ However, brain MRI abnormalities, including leukoencephalopathy, are absent in approximately half of NIID patients presenting with muscle weakness-predominant symptoms.^4^ This underscores the unmet need for an additional screening tool applicable across all NIID phenotypes. MNCV slowing emerges as a more prevalent characteristic when contrasted with MRI abnormalities. Within our cohort, 46 out of 50 NIID patients (92%) exhibited MNCV slowing (Table 4), even though 11 out of 50 NIID patients (22%) displayed no remarkable leukoencephalopathy on brain MRIs. Consequently, the diagnostic value of the MNCV criterion for identifying NIID becomes distinctly evident.

A limitation of our study is the proposed MNCV criterion alone does not allow distinguishing between NIID and other peripheral neuropathies. Patients with diabetes polyneuropathy or chronic inflammatory demyelinating polyneuropathy may also meet the criterion. Among NIID patients, MNCV of upper limbs and lower limbs rarely fell below 35 and 30 m/s, respectively. Therefore, markedly reduced MNCV below these thresholds might suggest an alternative diagnosis. It is however, imperative to combine the MNCV criterion with other clinical information, such as family history, clinical presentations and neuroimaging features, to attain high accuracy in diagnosing NIID.

In conclusion, abnormal nerve conduction velocities are highly prevalent among patients with NIID. Mild to moderately decreased MNCV in multiple nerves represents a significant electrophysiological hallmark of NIID. In patients with unexplained leukoencephalopathy, dementia, parkinsonism or muscle weakness, nerve conduction studies can be used as a preliminary screening tool. The proposed criterion can help identify individuals among suspected cases who warrant further genetic diagnosis for NIID. It also provides opportunities for future pathological studies to understand the underlying mechanism of NIID neuropathy. Additionally, the proposed criterion may aid in early and accurate diagnosis of NIID, enabling clinicians to provide early interventions for symptoms related to NIID.

## Supplementary Material

fcae221_Supplementary_Data

## Data Availability

The data that support the findings of this study are available from the corresponding author. Data will be shared on reasonable request and after ethical approval if requested by other investigators.
