# Supplementary material for: Nerve conduction features may serve as a diagnostic clue for neuronal intranuclear inclusion disease
Source: Brain Commun. 2024 Jun 26;6(4):fcae221. doi: 10.1093/braincomms/fcae221 (PMC11229697; doi:10.1093/braincomms/fcae221)
Supplement: fcae221_Supplementary_Data [file fcae221_supplementary_data.docx]

**Supplementary materials**

**Supplementary Table 1. The 183 genes in the targeted resequencing panel**

| *AARS2* | *CBS* | *EGR2* | *FBN1* | *HARS2* | *LARS2* | *NOTCH3* | *PYCR2* | *SLC46A1* | *WARS2* |
| --- | --- | --- | --- | --- | --- | --- | --- | --- | --- |
| *ABCC1* | *CLCN2* | *EIF2B1* | *FIG4* | *HBB* | *LMNB1* | *NUBPL* | *RARS* | *SNORD118* | *XPA* |
| *ABCC6* | *CNTNAP1* | *EIF2B2* | *FOLR1* | *HEPACAM* | *MAG* | *PEX1* | *RARS2* | *SOX10* | *XPC* |
| *ABCD1* | *COA8* | *EIF2B3* | *FOXC1* | *HEXA* | *MAN2B1* | *PIGT* | *REEP1* | *SPG11* |  |
| *ACP5* | *COL3A1* | *EIF2B4* | *FUCA1* | *HEXB* | *MANBA* | *PITX2* | *RNASEH2A* | *SUCLG1* |  |
| *ACTA2* | *COL4A1* | *EIF2B5* | *GALC* | *HIKESHI* | *MAPT* | *PLP1* | *RNASEH2B* | *SUMF1* |  |
| *ACVRL1* | *COL4A2* | *ENG* | *GAN* | *HMGCL* | *MARS2* | *POLG* | *RNASEH2C* | *SUOX* |  |
| *ADA2* | *COLGALT1* | *EPRS* | *GBA* | *HSPD1* | *MCOLN1* | *POLH* | *RNASET2* | *SURF1* |  |
| *ADAR* | *CSF1R* | *ERCC1* | *GBE1* | *HTRA1* | *MLC1* | *POLR1C* | *RNF113A* | *TARS2* |  |
| *AIMP1* | *CST3* | *ERCC2* | *GCDH* | *IARS2* | *MMACHC* | *POLR3A* | *RPIA* | *TGFBR2* |  |
| *AIMP2* | *CTC1* | *ERCC3* | *GFAP* | *IBA57* | *MPLKIP* | *POLR3B* | *RRM2B* | *TMEM106B* |  |
| *ALDH3A2* | *CTSA* | *ERCC4* | *GJA1* | *IDUA* | *MPV17* | *POLR3K* | *SAMHD1* | *TREM2* |  |
| *APLP1* | *CTSD* | *ERCC5* | *GJB1* | *IFIH1* | *MRM2* | *PRNP* | *SARS2* | *TREX1* |  |
| *APLP2* | *CYP27A1* | *ERCC6* | *GJC2* | *ISCA2* | *MRPL38* | *PROC* | *SCP2* | *TTR* |  |
| *APOE* | *CYP2U1* | *ERCC8* | *GLA* | *ITM2B* | *MTCL1* | *PROS1* | *SDHAF1* | *TUBB4A* |  |
| *APP* | *DARS* | *F2* | *GLB1* | *JAM3* | *MTHFD1* | *PROZ* | *SERPINC1* | *TYMP* |  |
| *ARSA* | *DARS2* | *F5* | *GRN* | *KARS* | *MTHFR* | *PSAP* | *SERPINE1* | *TYROBP* |  |
| *ASPA* | *DDB2* | *FA2H* | *GSN* | *KIF5A* | *NF1* | *PSEN1* | *SLC16A2* | *VARS2* |  |
| *AUH* | *DHFR* | *FAM126A* | *GTF2E2* | *L2HGDH* | *NFU1* | *PSEN2* | *SLC17A5* | *VHL* |  |
| *CA2* | *EARS2* | *FARS2* | *GTF2H5* | *LAMB1* | *NKX6-2* | *PSENEN* | *SLC2A10* | *VPS11* |  |

**Supplementary Table 2. Comparison of the electrophysiological features between the NIID patients with CNS-predominant symptoms and PNS-predominant symptoms**

| Nerves | Parameters | Mean ± SD or Number (%) | | p value* |
| --- | --- | --- | --- | --- |
|  |  | CNS- predominant  (N = 35) | PNS- predominant (N = 15) |  |
| Median nerve | MNCV (m/s) | 43.7 ± 4.9 | 46.3 ± 6.7 | 0.62 |
|  | CMAP (m/v) | 6.2 ± 2.6 | 7.4 ± 2.4 | 0.81 |
|  | F wave latency (ms) | 31.3 ± 3.0 | 33.3 ± 4.7 | 0.16 |
|  | Absent F wave | 1 (3.2%) | 2 (13.3%) |  |
|  | SNCV (m/s) | 42.3 ± 5.4 | 43.5 ± 7.1 | 0.13 |
|  | SNAP (μ/v) | 25.5 ± 15.3 | 31.8 ± 18.4 | 0.30 |
| Ulnar nerve | MNCV (m/s) | 45.5 ± 6.5 | 44.3 ± 6.0 | 0.64 |
|  | CMAP (m/v) | 7.3 ± 2.0 | 7.5 ± 2.1 | 0.89 |
|  | F wave latency (ms) | 31.9 ± 3.7 | 33.6 ± 3.9 | 0.90 |
|  | Absent F wave | 0 (0%) | 1 (6.7%) |  |
|  | SNCV (m/s) | 43.4 ± 5.2 | 43.0 ± 5.3 | 0.58 |
|  | SNAP (μ/v) | 23.2 ± 12.7 | 26.3 ± 13.3 | 0.41 |
| Peroneal nerve | MNCV (m/s) | 37.2 ± 5.1 | 38.2 ± 6.3 | 0.47 |
|  | CMAP (m/v) | 3.6 ± 1.9 | 3.3 ± 1.8 | 0.90 |
|  | F wave latency (ms) | 55.6 ± 7.4 | 57.5 ± 7.6 | 0.62 |
|  | Absent F wave | 7 (22.6%) | 3 (20.0%) |  |
| Tibial nerve | MNCV (m/s) | 34.9 ± 5.0 | 37.3 ± 5.9 | 0.65 |
|  | CMAP (m/v) | 7.4 ± 4.3 | 9.9 ± 5.6 | 0.30 |
|  | F wave latency (ms) | 57.6 ± 8.3 | 58.8 ± 5.4 | 0.34 |
|  | Absent F wave | 2 (6.5%) | 2 (13.3%) |  |
| Sural nerve | SNCV (m/s) | 40.6 ± 9.0 | 43.8 ± 6.4 | 0.32 |
|  | SNAP (μ/v) | 12.4 ± 6.4 | 12.7 ± 7.1 | 0.80 |
|  | Not evoked | 10 (32.2%) | 6 (40.0%) |  |

CMAP: compound muscle action potential; CNS: central nervous system; MNCV: motor nerve conduction velocity; NIID: neuronal intranuclear inclusion disease; PNS: peripheral nervous system; SD: standard deviation; SNCV: sensory nerve conduction velocity; SNAP: sensory nerve action potential

* Comparing between NIID patients with CNS-predominant symptoms and those with PNS-predominant symptoms using Student’s t test.

**Supplementary Table 3. The relationship between the disease duration at NCS exam and the percentages of the patients with abnormal nerve conduction findings in this cohort.**

| Disease duration at NCS exam (years) | Number of NIID patients | Patients with abnormal nerve conduction findings, N (%) |
| --- | --- | --- |
| 1≤ | 14 | 13 (92.9%) |
| 1-2 | 5 | 5 (100%) |
| 2-3 | 4 | 4 (100%) |
| 3-4 | 3 | 3 (100%) |
| 4-5 | 2 | 2 (100%) |
| 5-10 | 8 | 8 (100%) |
| >10 | 8 | 7 (87.5%) |
| Unknown | 5 | 5 (100%) |

NCS: nerve conduction studies

**Supplementary Table 4. Inconsistent involvement of the sensory and motor components of individual nerve in patients with neuronal intranuclear inclusion disease (NIID) in this study.**

| Patient number, N (%) |  | MNCV | |
| --- | --- | --- | --- |
|  | SNCV | Abnormal | Normal |
| Median nerve | Abnormal | 27 (54%) | 0 (0%) |
|  | Normal | 15 (30%) | 8 (16%) |
| Ulnar nerve | Abnormal | 25 (50%) | 5 (10%) |
|  | Normal | 12 (24%) | 8 (16%) |

Abnormal MNCV and abnormal SNCV are defined as values less than mean - 2 standard deviation of the 200 normal controls on the examined nerves or unable to evoke a response.

**Supplementary Figure 1.** (A) The relationship between motor nerve conduction velocities in the median nerves (median MNCV) and the sizes of the expanded alleles of *NOTCH2NLC* GGC repeats, and (B) the relationship between median MNCV and the age at exam in the patients with neuronal intranuclear inclusion disease (NIID) in this study.


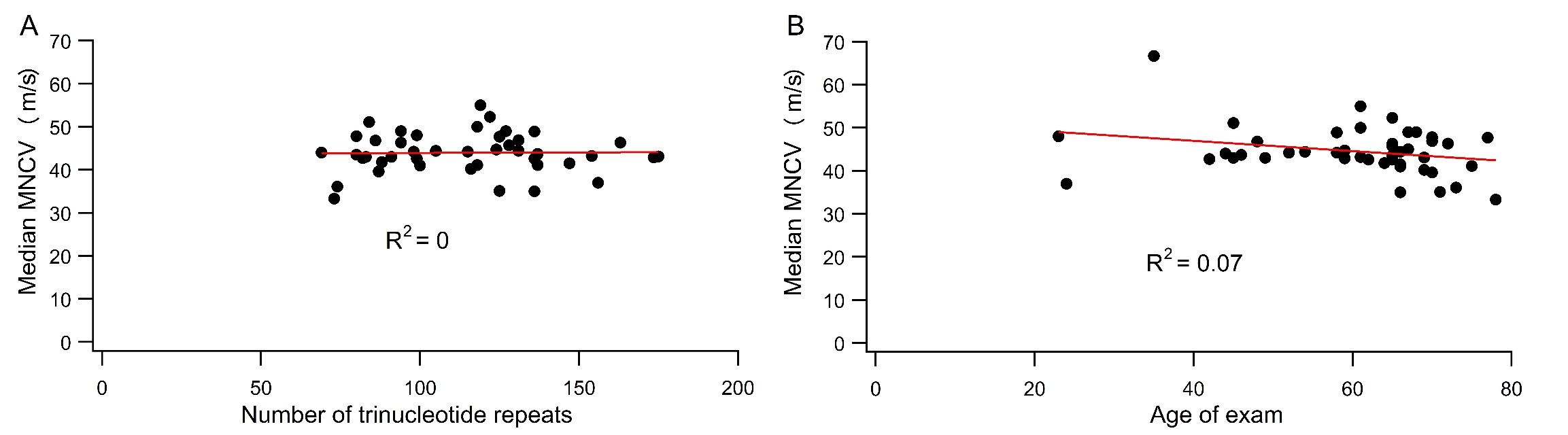


R² is a goodness-of-fit measure of the regression model to show the proportion of variance in median MNCV that can be explained by GGC repeat numbers or explained by age of exam.
